# Supplementary material for: Systematic review of contemporary interventions for improving discharge support and transitions of care from the patient experience perspective
Source: PLoS One. 2024 May 21;19(5):e0299176. doi: 10.1371/journal.pone.0299176 (PMC11108181; doi:10.1371/journal.pone.0299176)
Supplement: S4 Appendix — (DOCX) [file pone.0299176.s004.docx]

**S4 Appendix – Quality Appraisals**

**Cochrane-suggested risk of bias criteria for Effective Practice and Organization of Care reviews for randomized trials, non-randomized trials, and controlled before-after studies**

| **Citation and intervention group** | **Random sequence generation [1]** | **Allocation concealment [2]** | **Baseline outcomes similar [3]** | **Baseline characteristics similar [4]** | **Incomplete outcome data [5]** | **Knowledge of allocated interventions adequately prevented [6]** | **Protection against contamination [7]** | **Selective outcome reporting [8]** | **Other risks of bias [9]** | **Notes** |
| --- | --- | --- | --- | --- | --- | --- | --- | --- | --- | --- |
| LaBedz et al 2022  Transitional Care | **LOW** | **LOW** | **HIGH** | **LOW** | **LOW** | **LOW** | **LOW** | **LOW** | **HIGH** | *[5] About a 20% loss to follow-up but similar across groups; multiple sensitivity analyses employed to account for missing data.*  *[9] Limited fidelity of the implementation: Only 29% Navigator group participants received the intervention per protocol. Single-center study affects the generalizability. While the authors use a standardized measure, it is not a traditional patient experience measure, albeit a standardized one, and may not be specific enough or responsive enough for the intervention.* |
| Noël et al., 2022  Care Coordination | **LOW** | **LOW** | **HIGH** | **LOW** | **LOW** | **LOW** | **LOW** | **LOW** | **LOW** | *[3] Patient experience measures were imbalanced at baseline, with non-coached clinics reporting more hassles. This could lead to increased opportunity for hassles to improve over time in non-coached clinics, biasing results in favor of non-coached clinics. Since the study did not include a “usual care” control group, one does not know whether the observed improvements were the result of the intervention versus secular trends unrelated to the interventions. Also, pre and post groups were different. [4] Participation was not mandated, participating clinics might have been more highly engaged than average, such that findings may not generalize to VA primary care nationwide. Different groups pre and post, yet the analysis was adjusted. [9] Limited comparisons - don't need Bonferroni.* |
| Zulman et al., 2019  Care Coordination | **LOW** | **LOW** | **UNCLEAR** | **LOW** | **LOW** | **LOW** | **HIGH** | **LOW** | **HIGH** | *[2] Not clearly reported in this article but clearer in paper describing the RCT (PMID: 29868706).*  *[3] No baseline outcomes.*  *[4] Different groups pre and post.*  *[6] Self-reported surveys.*  *[7] Patients may not distinguish the intervention from the support previously provided (control).*  *[9] Not all patients randomized to the intervention received the intervention. Multiple comparisons - no correction.* |
| Dorr et al., 2016  Care Coordination | **LOW** | **LOW** | **LOW** | **LOW** | **LOW** | **LOW** | **LOW** | **LOW** | **HIGH** | *[3] Different groups pre and post.*  *[4] Different groups pre and post.*  *[6] Statistician blinded - self-report.*  *[9] Use of P value threshold of P < .10 and all significant changes identified ranged between P > 0.5 and P < .10. Small number of clusters. Both selection and respondent bias were evident; although a randomized stratified sample of patients was created, those motivated to respond in the pre- and post-periods were different. Although statistically attempted to account for these differences, some bias may remain. Multiple comparisons - no correction.* |
| Nembhard et al., 2020  Care Coordination | **HIGH** | **HIGH** | **LOW** | **LOW** | **LOW** | **HIGH** | **LOW** | **LOW** | **HIGH** | *[2] Cluster-allocated but controlled before/after.*  *[3] Not clearly reported but seems similar in figure.*  *[4] Some differences but adjusted for in analyses.*  *[7] Cluster-allocated.*  *[9] Centers affiliated with one organization in one state; selection bias - those who selected to participate differed from those who did not. Intervention not consistently delivered as physicians elected to contact for further support. Limited comparisons - don't need correction.* |
| van Eck et al., 2018  Discharge Support | **LOW** | **UNCLEAR** | **UNCLEAR** | **LOW** | **LOW** | **HIGH** | **LOW** | **LOW** | **HIGH** | *[2] Not specified but surgeons blinded to group assignment.*  *[3] No baseline outcomes.*  *[6] Patients not blinded.*  *[7] In same clinic but nature of intervention limits contamination.*  *[9] Single practice with surgeons. Single setting. Only about 90 patients for the pre- and post-intervention. Limited follow-up (only two weeks of post-assessment). Multiple comparisons - no correction.* |
| Griffey et al., 2015  Discharge Support | **HIGH** | **HIGH** | **UNCLEAR** | **LOW** | **HIGH** | **HIGH** | **HIGH** | **LOW** | **HIGH** | *[1] Allocated based on odd or even number in medical record number.*  *[3] No baseline outcomes.*  *[4] Differences in race but adjusted for in analyses.*  *[5] 127 completed the protocol out of 212 or 196 randomized for the intervention and control group respectively, i.e. 40% loss for the intervention group.*  *[7] Some level of contamination may have occurred as same nurses.*  *[9] One site, convenience sampling before randomization. Multiple comparisons - no correction.* |
| Chan et al., 2015  Discharge Support | **LOW** | **LOW** | **UNCLEAR** | **LOW** | **LOW** | **LOW** | **HIGH** | **LOW** | **HIGH** | *[1] Unclear in paper but clearer in main trial paper (PMID: 25285540).*  *[2] Unclear in paper but clearer in main trial paper (PMID: 25285540).*  *[3] No baseline outcomes.*  *[6] Medical teams were not blinded, but RAs collecting data at 30 days were blinded to randomization status.*  *[7] Some level of contamination may have occurred given patient-level randomization.*  *[9] Single site. Limited follow-up (30 days). Multiple comparisons - no correction.* |
| Centrella-Nigro et al., 2017  Discharge Support | **HIGH** | **HIGH** | **HIGH** | **HIGH** | **UNCLEAR** | **UNCLEAR** | **HIGH** | **LOW** | **HIGH** | *[3] Differences noted that are not adjusted for.*  *[4] Not reported.*  *[5] Numbers not clearly specified so % reporting is unclear.*  *[7] Some risks: units of the same hospital. Those in control group may also have received this very basic intervention.*  *[9] Control and experimental units are different medical units. Although two control groups were used, their inferential results were computed in models apart (i.e., no difference-in-differences). Analysis is really only within-group, not a combined within/between group. Fidelity check consisted of self-documentation, not behavioral assessment of how well was it delivered. Multiple comparisons - no correction.* |
| Cancino et al., 2017  Discharge Support | **HIGH** | **HIGH** | **HIGH** | **HIGH** | **HIGH** | **LOW** | **HIGH** | **LOW** | **HIGH** | *[3] Not reported.*  *[4] Not reported.*  *[5] Low response rate.*  *[6] Self-report survey administered by 3rd party.*  *[7] Some patients in same unit may have gotten components of the intervention.*  *[9] Non-comparable samples and no adjustment for possible confounders. Findings may not be transferable to hospital with higher baseline patient experience discharges. No adjustment and non-randomized groups, may have selection bias with those selected for intervention from within unit. Multiple comparisons - no correction.* |

**National Heart, Lung, and Blood Institute’s Quality Assessment Tool for Observational Cohort and Cross-Sectional Studies**

| **Citation and intervention group** | **Quality** | **Research question** | **Clear population definition** | **Participation rate** | **Uniform eligibility** | **Sample size justification** | **Exposure before outcome** | **Sufficient timeframe** | **Different exposure levels** | **Exposure measures** | **Repeat exposure assessment** | **Outcome measures** | **Blinding of outcome assessors** | **Follow-up rate** | **Statistical analysis** | **Notes and other limitations** |
| --- | --- | --- | --- | --- | --- | --- | --- | --- | --- | --- | --- | --- | --- | --- | --- | --- |
| Jones et al., 2019  Care Coordination | **FAIR** | **YES** | **YES** | **NR** | **NO** | **NO**  (but large multi-site study) | **YES** | **YES** | **NA** | **NA** Intervention | **NA**  Single intervention | **YES** | **NA**  Self-report measures | **NO** | **YES** | *Site-specific criteria for intervention eligibility. No baseline outcome measured. Outcomes measure does not address homeless-specific concerns and service needs.*  *No correction for multiple comparisons.* |
| Tung et al., 2018  Care Coordination | **FAIR** | **YES** | **YES** | **NR** | **YES** | **NO**  (but large multi-site study) | **YES** | **YES** | **NA** | **NA** Intervention | **NA**  Single intervention | **YES** | **NA**  Self-report measures | **NO** | **YES** | *Patient baseline measures assessed 13 months after intervention began.*  *All analyses at clinic level with inability to follow individual patients over time. No correction for multiple comparisons.*  *Potential selection bias: only 13 of the 24 clinics that completed baseline surveys were included in the analyses as they also completed the post-intervention ones.* |
| Nguyen et al., 2020  Care Coordination | **FAIR** | **YES** | **YES** | **NR** | **YES** | **NO**  (but large multi-site study) | **YES** | **YES** | **NA** | **NA** Intervention | **NA**  Single intervention | **YES** | **NA**  Self-report measures | **NR** | **YES** | *External comparators with unknown practice patterns.*  *No correction for multiple comparisons.*  *Although adjusted models were used, the study was unable to control for a wide variation in the size, structure, and composition of multidisciplinary teams established following the transition from traditional practice organization to team-based care.* |
| Schreiter et al., 2021  Discharge Support | **FAIR** | **YES** | **YES** | **YES**  (for intervention) | **NO** | **NO** | **YES** | **YES** | **NA** | **NA** Intervention | **NA**  Single intervention | **YES** | **NA**  Self-report measures | **NR** | **NO**  No adjustment for Press Ganey | *Historical controls (although matching at patient level).*  *No correction for multiple comparisons. Single site. No assessment of baseline outcomes.* |
| CD = Cannot Determine; NR = Not Reported; NA = Not Applicable. | | | | | | | | | | | | | | | | |

**National Heart, Lung, and Blood Institute’s Quality Assessment Tool for Before-After (Pre-Post) Studies With No Control Group**

| **Citation and intervention group** | **Quality** | **Research question** | **Eligibility criteria** | **Representative of clinical population** | **All eligible enrolled** | **Sample size** | **Intervention clearly described** | **Outcome measures** | **Blinding of outcome assessors** | **Follow-up rate** | **Statistical analysis** | **Multiple outcome measures** | **Group-level interventions** | **Notes and other limitations** |
| --- | --- | --- | --- | --- | --- | --- | --- | --- | --- | --- | --- | --- | --- | --- |
| Thum et al., 2022  Discharge | **FAIR/ POOR** | **YES** | **NO** | **CD** | **CD** | **YES** | **YES** | **YES** | **NA**  (Self-report measures) | **CD** | **YES** | **NO** | **NO** | *Eligibility criteria not clearly described.*  *Single pretest (although long preintervention period).*  *Different pre- and post- groups at the patient level, without establishing stable baseline through multiple measurement time points.*  *No report on the numbers of surveys used for the analysis, only those mailed.*  *No correction for multiple comparisons.* |
| March et al., 2022  Discharge | **FAIR/ POOR** | **YES** | **YES** | **CD** | **NO** | **NO** | **YES** | **YES** | **NA**  (Self-report measures) | **CD** | **YES** | **NO** | **NO** | *Limited information on sample, including size of pre-group.*  *Risks of selected findings: two different designs for two outcomes (pre-post for the primary outcome, case control for the secondary).*  *Pre/post in different groups with lack of multiple measurement points to ensure stable baseline.*  *Intervention not delivered to all eligible patients.*  *No correction for multiple comparisons.* |
| CD = Cannot Determine; NR = Not Reported; NA = Not Applicable. | | | | | | | | | | | | | | |

**References**

Cancino RS, Manasseh C, Kwong L, et al. Project RED impacts patient experience. *J Patient Exp* 2017;4(4):185-90. doi: 10.1177/2374373517714454 [published Online First: 20170616]

Centrella-Nigro AM, Alexander C. Using the teach-back method in patient education to improve patient satisfaction. *J Contin Educ Nurs* 2017;48(1):47-52. doi: 10.3928/00220124-20170110-10

Chan B, Goldman LE, Sarkar U, et al. The effect of a care transition intervention on the patient experience of older multi-lingual adults in the safety net: results of a randomized controlled trial. *J Gen Intern Med* 2015;30(12):1788-94. doi: 10.1007/s11606-015-3362-y

Dorr DA, Anastas T, Ramsey K, et al. Effect of a pragmatic, cluster-randomized controlled trial on patient experience with care: The Transforming Outcomes for Patients through Medical Home Evaluation and Redesign (TOPMED) study. *Med Care* 2016;54(8):745-51. doi: 10.1097/mlr.0000000000000552

Griffey RT, Shin N, Jones S, et al. The impact of teach-back on comprehension of discharge instructions and satisfaction among emergency patients with limited health literacy: A randomized, controlled study. *J Commun Healthc* 2015;8(1):10-21. doi: 10.1179/1753807615y.0000000001

Jones AL, Hausmann LRM, Kertesz SG, et al. Providing positive primary care experiences for homeless veterans through tailored medical homes: The Veterans Health Administration's homeless patient aligned care teams. *Med Care* 2019;57(4):270-78. doi: 10.1097/mlr.0000000000001070

March KL, Peters MJ, Finch CK, et al. Pharmacist transition-of-care services improve patient satisfaction and decrease hospital readmissions. *J Pharm Pract* 2022;35(1):86-93. doi: 10.1177/0897190020958264 [published Online First: 20200918]

Nembhard IM, Buta E, Lee YSH, et al. A quasi-experiment assessing the six-months effects of a nurse care coordination program on patient care experiences and clinician teamwork in community health centers. *BMC Health Serv Res* 2020;20(1):137. doi: 10.1186/s12913-020-4986-0 [published Online First: 20200224]

Nguyen KH, Chien AT, Meyers DJ, et al. Team-based primary care practice transformation initiative and changes in patient experience and recommended cancer screening rates. *Inquiry* 2020;57:46958020952911. doi: 10.1177/0046958020952911

Noël PH, Barnard JM, Leng M, et al. The Coordination Toolkit and Coaching project: cluster-randomized quality improvement initiative to improve patient experience of care coordination. *J Gen Intern Med* 2022;37(1):95-103. doi: 10.1007/s11606-021-06926-y [published Online First: 20210609]

Schreiter NA, Fisher A, Barrett JR, et al. A telephone-based surgical transitional care program with improved patient satisfaction scores and fiscal neutrality. *Surgery* 2021;169(2):347-55. doi: 10.1016/j.surg.2020.09.015 [published Online First: 20201020]

Thum A, Ackermann L, Edger MB, et al. Improving the discharge experience of hospital patients through standard tools and methods of education. *J Healthc Qual* 2022;44(2):113-21. doi: 10.1097/jhq.0000000000000325

Tung EL, Gao Y, Peek ME, et al. Patient experience of chronic illness care and medical home transformation in safety net clinics. *Health Serv Res* 2018;53(1):469-88. doi: 10.1111/1475-6773.12608 [published Online First: 20170530]

van Eck CF, Toor A, Banffy MB, et al. Web-based education prior to outpatient orthopaedic surgery enhances early patient satisfaction scores: a prospective randomized controlled study. *Orthop J Sports Med* 2018;6(1):2325967117751418. doi: 10.1177/2325967117751418 [published Online First: 20180126]

Zulman DM, Chang ET, Wong A, et al. Effects of intensive primary care on high-need patient experiences: survey findings from a Veterans Affairs randomized quality improvement trial. *J Gen Intern Med* 2019;34(Suppsl 1):75-81. doi: 10.1007/s11606-019-04965-0
