# Supplementary material for: Systematic review of contemporary interventions for improving discharge support and transitions of care from the patient experience perspective
Source: PLoS One. 2024 May 21;19(5):e0299176. doi: 10.1371/journal.pone.0299176 (PMC11108181; doi:10.1371/journal.pone.0299176)
Supplement: Attachment — Submitted filename: Response to Reviewers PLOS One TOC paper.docx [file pone.0299176.s005.docx]

We are grateful to the reviewers for the wonderful feedback. We reply below (in blue) on a point-by-point basis.

Reviewer #1:

The authors reviewed contemporary literature describing the impact of care coordination, discharge support, and transitional care activities on patient experiences as assessed by formal patient-reported experience measures. The Reviewer thinks that this is a unique report and worthy.

Thank you!

The papers enrolled in this review were ultimately limited in number and varied in the type and degree of bias, which may have prevented them from drawing impactful conclusions. However, the Reviewer appreciates that it is significant in that it clarifies that such a situation exists because no systematic review focused on the impact that care coordination, discharge support, or transitional care activities have on the patient’s experience with care.

We do believe we synthesized the state-of-the-science in this unaddressed domain.

Reviewer #2:

Thank you for the opportunity to review this work. I greatly appreciate the goal of synthesizing the transitional care literature. It's variability and breadth is difficult to navigate.
I believe the overarching theme of this manuscript is that there has not been much/enough work directly evaluating the patient experience related to transitions of care (TOC) services. The authors worked to capture the findings of a variable cross-section of the TOC literature specifically related to this outcome. This manuscript highlights a gap in the current literature and seems to be quite comprehensive. I believe that this is a valuable addition to the literature that would benefit from revision to enhance readability. Specific feedback can be found below:

We are grateful about these comments.

Abstract:
- Is it possible to make the aim more concise? Can the domains of care coordination and discharge support be grouped together under a single heading (e.g. TOC)? This would bring the focal point to the process, in general, recognizing that there are many different features/components that this may encompass. This modification would likely also impact the title.

Insightful comment. We agree that the title could be more concise. and changed the title to be more concise by removing the care coordination component that is partly implicit.

The title now reads:

*Systematic review of contemporary interventions for improving discharge support and transitions of care from the patient experience perspective*

In the abstract and full paper, we believe the three distinction topics of discharge support, care coordination, and transitional activities are important to maintain. This wording is consistent with the three differentiated inclusion categories, matching the three-level operational definitions provided in the eligibility part of the Methods section, and subsequently the full-text results organized around these categories.
- Line 58: recommend a comma after "journals"

We made this edit.
- Line 58: consider adding "published" between "studies" and "in"

We made this edit.
- Line 58: consider adding "focused" before "on"

We made this edit.
- Line 62: remove "finally"

We removed this word.
- consider removing the number of RCT from the abstract or summarizing with the report of the 16 studies (e.g. "16 [10 RCT]"....

In the abstract, we made numerous changes in the results section to improve readability, including reducing the content within parenthesis as recommended and splitting one sentence into two. We kept the report of the number of RCTs, including the last sentence of the abstracts’ conclusion (need for an improved body of evidence).

- consider removing the clarifications in parentheses from the abstract to improve readability. This would necessitate improving the phrasing for "enhanced supports" in line 64

We removed several parentheses keeping only those that support rapid, illustrative information. The phase on “enhanced supports” is complemented by another one stating what were the “simpler supports”.

- Lines 65-66: does "effectiveness" relate to the intervention or impact on patient experience? Please clarify.

We have clarified this point.

- Line 70: it is unclear what the significance of "only for one patient experience item" means without the context of the tool used to assess the experience

The entire phrase was reframed to be more concise.

- Line 71: "out of challenges with implementation fidelity" is unclear

We reframed this sentence to improve clarity. As not uncommon on pragmatic trials and conditions, the protocoled intervention is not always delivered according to plan, which happened in this study, and can affect the results, and thus important to mention.

- Line 72: It is unclear what differentiates "enhanced supports" from "simpler improvement approaches"

We had a statement in the preceding results describing simpler approaches, and were comparing the more complex enhanced supports.

- Within the conclusion there is a need stated that is not represented by the rest of the abstract. In the absence of background information to demonstrate this need, it may make sense to state that the patient experience is important to the care process that there's a gap in the literature in this area.

We re-worded the last sentence for improved clarity.

Background
- Line 85: consider modifying "valuable"

We replaced this word with the word “key”

- Line 86: please define "supports"

We revised this sentence and report to “care coordination and discharge planning activities”

- Line 89: remove "to"

We removed this word.

- Line 90: I don't think the examples within the parentheses are needed
We have removed the examples, as suggested.

- Line 96: remove "increasingly"

We have removed this word, as suggested.

- Lines 101-102: remove "reflecting patient experience of care" since it was defined above

Great comment! We have removed this statement, as suggested.

- Line 102: consider "calculating" instead of "computing"

We have replaced “computing” with “calculating, as suggested..

- Line 104: consider whether "increasingly" is needed here

We have removed this word, as suggested.
- Line 106: consider grouping care coordination and discharge support or making it clear that they are being linked to care transitions

As noted above regarding the abstract, we believe it is important to maintain these three categories, which is consistent with the operational definition in the Methods section.
- Line 108: please define "supported discharge"

We have added “coordinated transfer of care from hospital to patient’s home” to clarify “supported discharge.”
- Line 109: add a comma after coordinators

We have added the comma, as suggested.
- Lines 107-110: should there be a citation for the first sentence?

We agree and have added two references.
- Line 111: consider removing "of these"

We have removed this word.
- Line 112: the example within the parentheses is not needed if a similar example is provided in the next sentence

We agree and have removed this example.
- Line 117: consider merging this paragraph with the previous paragraph

We have merged the paragraphs, as suggested.
- Line 118: consider modifying to "on patient experiences with care"

We did modify this sentence, as suggested.
- Line 120: consider modifying "synthesized as outcomes"

We have revised the wording to use “reviewed.”
Methods
- Line 129: remove "the" after "to improve"

We have removed “the” as suggested.
- Line 131: it may be more clear to add "on the patient experience" or something similar

We have added the phrase, as suggested.
- Line 137: it may be clearer to say, "full texts available, report patient experience as a primary outcome, have no more than two primary outcomes,...."

We have simplified, as suggested.
- Line 141: suggest removing the parentheses and stating, "assessments including...."

We have made the edit suggested.
- Line 147: does "participants" mean participating locations or service providers? Please clarify.

We kept the term “participants” to align with the known acronym PICO, In this case, the meaning is a wide range of service providers and staff.

- Line 150: please define "proxy respondents"

Now have revised this sentence to state: “could be the patient, family/informal caregivers, or any of those as proxy respondents on the patient’s behalf.”

- Line 173: consider rephrasing (...searched in the time since be coming peer reviewed (2020)...."

We have revised the sentence to state: “The journal *NEJM Catalyst Innovations in Care Delivery* was searched since January 2020, when the journal became peer reviewed.”

- Line 181: consider modifying "involved" to something like "engaged" or "consulted"

We have revised this sentence to use the word “engaged.”
- Line 183: consider substituting "including" for "such as"

We have made this edit.
- Line 192: citation for EPOC needed

We have added this citation.
- Line 194: citation needed for NHLBI tools

We have added this citation.
- Line 199: no comma needed after "design"

The comma was removed.

Results
- It would be helpful to have more information in the text about the patient experience measure used in each section.

This was a challenging component in the write up of this review. A number of different measures and/or metrics were used, within and across studies. In the initial drafts of the manuscript, we had more on this information within the text; however it became burdensome to read and, paradoxically, did not add clarity. So, at that point, we designed the tables to include a matrix-based display of the measures and metrics used, and results for each of them. We believe this approach allows the reader to more easily comparable results across studies. Albeit not ideal, we were more satisfied with that display in the tables than the text description - for an already long and nuanced text-based reporting of the results.

- Line 219: the information in the parentheses does not clarify the "supports" designation. What are "over and beyond models"?

We now clarify that we mean additional care coordination supports beyond those provided as standard in PCMHs. As this is an introductory paragraph to the scope of the research synthesized, more details on which type of supports are provided in the following paragraphs.

- Lines 234-235: the description of the study is unclear, specifically, "...used QI facilitation (e.g. coaching) on the site's use of a...."

We revised the sentence and explain the use of “QI facilitation such as coaching by centralized improvement experts to support the site’s use of a care-coordination improvement toolkit”.

- Line 235: it may be clearer to remind the reader that the outcome that improved was patient experience

We agree and have made this edit.

- Lines 235-236: more detail is needed to understand what makes up the "more resource-intensive arm" and what this means for the result.

We have now clarified that this refers to using coaching facilitation.

- Line 239: to clarify - are these care coordination items patient-experience outcomes?

Yes, these items reflect the patient experience perspective, and we have clarified that point.

- Lines 239, 250-251: the details of this study are unclear in the text ("encouraged the selection"). Does this refer to study design or outcomes.

We revised the sentence to state that “The remaining RCT focused on encouraging the selection of high-value care-coordination QI goals…” to allude to the nature of this type of RCT: a so-called encouragement trial, whose description in the text is now more aligned with the nature of this trial.

- Line 268: write out "and"

We have made this edit.

- Line 287: consider including a short list of examples instead of the information in parentheses

We removed the parentheses and provide the example as part of the sentence. More examples can be found in the tables with the more detailed results.

- Line 287: consider removing "in turn"

We have removed these words, as suggested.

- Line 289: remove the semi-colon and create two distinct sentences.

We removed the semi-colon and created two setnences, as suggested.

- Line 303: the information within the parentheses is redundant with the previous sentence

We agree, and have removed this information.

- Lines 300-308: this is the best description within the narrative. It is clear and concise.

Thanks!

Discussion
- Lines 319-320: sometimes the author speaks of two areas of review (care coordination and discharge support) and in some places three. I suggest being consistent throughout.

Awesome commentary. We have addressed this issue and also added information about the transitional approach. Even though only one study was specifically focused on this topic, it is the third category we do include and address. We added that topic to the first paragraph of the Discussion too for consistency.

- Line 323: add a space between "usual" and "care"

We have made this edit.

Lines 324-325: Based on the previous sentence, should the findings of both the 7 and 8 studies be described in this sentence? Also, the third outcome (TOC) is left out of this paragraph.

As noted above, we added the TOC outcome. The first paragraph is becoming long, so we added a new paragraph to address the specific branches of this body of literature in more detail.

- Lines 333-339: Is there a way to make this more general to the study as a whole instead of focusing on details of a single study previously provided within the results?

We believe this study is illustrative of a more generable point. The conditions at given contexts may affect the results of controlled studies using ‘standard’ supports as controls. Hence, we were trying to provide a caution. However, providing context to that discussion point resulted in some level of repetition.

- Line 344: the last phrase of this sentence may be unnecessary

We have removed that phase.

- Line 345: consider modifying "here" to something like "within this review."

We have made this edit, as suggested.
- Line 345: consider updating the phrase "most solid evidence" and speak to rigor or level of evidence

We reworded this sentence to address the level of evidence; the issue of rigor is then addressed by the “well conducted”.

- Line 354: consider removing "often"

We have removed this word, as suggested.
- Line 356: the importance of the use of percentile ranks is unclear.

We have clarified the text as follows: “*Reporting results in more than one metric of a patient experience measure can be important, especially for percentile ranks in pre-post studies. This is because percentile rank metrics accounts for the performance of peer providers, it partly addresses the lack of a control group or the use of historical ones*.”
- Line 359: clarify "in more than one metric of a patient experience"

This issue was addressed when we revise the sentence noted above.
- Line 363: consider removing "at varying levels of available resources"

We have removed this phrase.
- Line 366: consider removing information within the parentheses.

We have removed this information, as suggested.
- Lines 371-379: this information sounds like it should go in the results section instead of the discussion

We understand the merits of the comment, considering the sentence as standalone pieces of information. However, we believe the sentences are helpful to establish a link between the results from a previous review related to the topic, what we found, and the specific needs for further research. We believe the last point of the paragraph is supported when these sentences are retained.

- Line 382: is "active ingredients" appropriate language within this study area?

While we recognize the terminology has origins in pharmacology and related fields of research, it is increasingly used within a broader range of research, including health services. So, we believe it can be appropriate for this context too.

- Lines 380-382: how does this relate to the assessment of patient experience?

We agree that is not necessarily specific to patient experience issues. However, the sentence provides suggestions of methodological approaches for strengthening the body of evidence, which fit the current, synthesized state of knowledge on the effectiveness of these interventions assessed from the patient experience perspective.

- Lines 393-395: citation needed

Good point. Two references were added.
- Lines 412-414: the second half of this sentence may not be appropriate for the limitations section

We agree, and this was removed.
- Overall, I am missing a "take home point" from the discussion. It sounds like we don't really have enough data, need to focus studies on the patient experience, etc., but it would be helpful to have the large points summarized in one specific area.

This is a great point. We now introduce a final summary paragraph with key take aways:

“*In a synthesis, the studies on the impact of improvement interventions related to care coordination, discharge support and care transitions on patient experience measures achieved mixed to null improvements, and the results are frequently not generalizable. To further advance this body of knowledge, strengthening activities might include developing cross-organizational studies, assuring a wider use of controlled designs, improving the implementation fidelity, and even further engaging the patient experience perspective into intervention development beyond its assessment.^58^*”

Conclusion
- Line 427: the second of this half is not needed

The literature was imbalanced in terms of methodological strengths. Hence, we believe it is important to point out which types of study weaknesses were most common, including the common feature of single-site studies.

- Lines 428-431: this seems redundant with previous sections and can be removed from conclusions

We agree that it addresses issues previously addressed in more detail, notably in the Discussion. However, we believe this is an important take way we need to summarize as a remark in the Conclusion.

- Line 434: consider modifying "improvement" to "impact on" to remove the assumed directionality of the outcome. There is potential that some interventions may worsen the patient experience.

Our sentence was oversimplistic. We re-worded to read: “*There is a need to strengthen the body of evidence on the impact of health-service improvement activities assessed from the patient experience perspective, especially via enhanced patient discharge support and transitional approaches.”*

Figure 1
- Clear and easy to follow

Thanks
- Consider adding the most common reasons for exclusion to the figure
Figure 1 provides specific reasons for exclusion at the full-text screening level, which is the level at which the PRISMA statement and flowchart recommend the report of the exclusion reasons.
Miscellaneous
- The summary tables are clear with appropriate details.

Thanks!

- It may be helpful to have a more lengthy description of the measures of patient experience.

This is an understandable request. However, a description of the measures would be quite lengthy. Our focus is the effectiveness of improvement interventions. While we do not provide a lengthy description of the measures (and rather focus on the improvement activities and results), we report whenever any adapted or modified use is made by the authors of the intervention studies – for example in table 2.

**Journal Requirements:**

When submitting your revision, we need you to address these additional requirements.

1. Please ensure that your manuscript meets PLOS ONE's style requirements, including those for file naming. The PLOS ONE style templates can be found at

https://journals.plos.org/plosone/s/file?id=wjVg/PLOSOne_formatting_sample_main_body.pdf and

https://journals.plos.org/plosone/s/file?id=ba62/PLOSOne_formatting_sample_title_authors_affiliations.pdf

We now address these requirements, including the naming of the supplementary files.

2. Thank you for stating the following financial disclosure:

"Tiago S. Jesus completed part of this work and Manrui Zhang the totally of this work under a grant from the National Institute on Disability, Independent Living, and Rehabilitation Research (NIDILRR; 90ARHF0003). NIDILRR is a center within the Administration for Community Living (ACL), U.S. Department of Health and Human Services (HHS). The contents of this publication do not necessarily represent the policy of NIDILRR, ACL, or HHS, and the reader should not assume endorsement by the U.S. federal government. Dr Jesus initiated this work with the Northwestern University’s affiliation (under the grant support mentioned) and the work was completed with The Ohio State University’s affiliation."

Please state what role the funders took in the study. If the funders had no role, please state: "The funders had no role in study design, data collection and analysis, decision to publish, or preparation of the manuscript."

If this statement is not correct you must amend it as needed.

Please include this amended Role of Funder statement in your cover letter; we will change the online submission form on your behalf.

All the above is now addressed in the resubmission.

3. Please provide a complete Data Availability Statement in the submission form, ensuring you include all necessary access information or a reason for why you are unable to make your data freely accessible. If your research concerns only data provided within your submission, please write "All data are in the manuscript and/or supporting information files" as your Data Availability Statement.

Executed as described.

4. Please include captions for your Supporting Information files at the end of your manuscript, and update any in-text citations to match accordingly. Please see our Supporting Information guidelines for more information: http://journals.plos.org/plosone/s/supporting-information.

This was now included.
